# Round the Hospitals

**Published:** 1916-06-17

**Authors:** 


### ROUND THE HOSPITALS.

THE First British Ambulance Unit for Italy, which has been at work since the summer of 1915, having started with a hospital of sixty beds and a fleet of twenty-six cars, was recently visited by the King of Italy and Prince Arthur of Connaught, who expressed themselves as being very pleased with the hospital. So successful has been its work and so much needed its services that new wards have been organised and equipped, and accommodation is now provided for 100 patients. Miss Price, the assistant matron at Paddington Infirmary, is the head sister at the hospital, and the staff of sisters and nurses has been much strengthened. The British Committee have been fortunate in receiving many gifts of hospital clothing, bedding, and necessaries, so that the hospital is stated to be a very comfortable one, whilst with the competent staff sent out by the committee the nursing cannot fail to be of the best.

WE are indebted to the matron of the Ramsgate General Hospital for the account of an interesting ceremony which took place at that hospital at the end of May on the occasion of the laying of the foundation-stone of the hospital's new sterilising room, the estimated cost of which is £200. This room and its fixtures, with the exception of the dressings steriliser, given by a resident twelve months ago and costing £60, are the gifts of Colonel and Mrs. George Winch and Mrs. Arthur Winch, the parents and aunt of Lieutenant Ronald Winch, who was accidentally killed by a sentry in Ramsgate last year. Lieutenant Winch, who was severely wounded in the abdomen, was brought to the hospital late one night and operated upon immediately, but he died on the second day after his admission. By the family's wish the ceremony of laying the stone was a quiet one. It was performed by Mrs. Arthur Winch; the vicar of the parish, who is also vice-president of the hospital, said a prayer, and short speeches were made by the president of the hospital, Mr. Charles Murray Smith, the chairman of the executive committee, and the treasurer. The inscription on the foundation-stone is as follows: "This sterilising chamber was built by Lieut.-Col. and Mrs. G. B. Winch in memory of their beloved and only child, Ronald Bluett Winch, who died in the service of his country at Ramsgate, 18th April, 1915, aged 20 years." The addition of the sterilising room makes the operation theatre unit at this hospital complete, and the late Lieutenant Winch's name will thus for ever be associated with the perfecting of this essential portion of an up-to-date hospital.

THE competent hospital housekeeper devotes much time and attention to the organisation of the store-room. This subject was dealt with from certain aspects in our issue of June 3, page 221. An important rule to observe is that no quantity, however small or however large, should be taken out of stock without a memorandum (which may be of the simplest description) being filed to record the transaction. At the end of every week these files should be emptied, the amount added up, and the totals recorded in a book kept for that purpose. The quantity used week by week will enable the average consumption over any given period to be reckoned out. In renewing stock from time to time these totals of the quantities consumed form a sure guide for the amount required, the quantity kept in stock being made up to the original weight or number. For instance, if three boxes of tea be kept in stock, the order for renewal would be given when two boxes had been consumed, and two boxes would be ordered. If 3 cwt. of sugar can be stored, the order for more would be given when two had been consumed, and the more regular the consumption the greater the satisfaction of the housekeeper. The original quantities stocked being entered in a stock-book and forming a separate item in the accounts, the average daily expenditure of the household should be calculated solely on the basis of what is withdrawn from stock. It is failure to grasp this elementary principle of the store-room which leads to waste and muddle in the use of stores.

MATRONS and sisters, for whom the question of financial provision for members of the nursing profession in their old age must always have importance, will be interested in the twenty-ninth annual report and accounts of the Royal National Pension Fund for Nurses, which has just been issued. The strength of the Fund is apparent from the fact that the reserve fund stands at £102,666, the total premiums received in 1915 were £120,458, and the invested funds amount to over two million pounds. The total number of nurses drawing annuities on December 31, 1915, was 2,059, the amount paid on this account being £54,500, or an average annuity of slightly over £26 9s. The Junius S. Morgan Benevolent Fund, which is the Benevolent branch of the Pension Fund, augments small pensions in deserving cases, about half the income of the Benevolent Fund being used for this purpose. The Fund's Emergency Committee, of which Miss Haughton, the matron of Guy's Hospital, is chairman, decides cases of urgent need. The annual meeting of the Pension Fund will take place on Thursday, June 22, at 3 P.M., at the Royal Society of Arts, John Street, Adelphi.

*** It is our invariable practice to pay for all items of news which we may publish. The minimum payment is 5s. Paragraphs of about twenty lines in length—that is to say, of 150 words or less—are preferred. In this section during the war we propose to give 10s. to the correspondent who may send us in any week the best incident connected with hospital life and work. In this way we hope to afford to all practical aid and encouragement; while those who reciprocate will be able to render us invaluable service. Contributions should be addressed to The Editor, THE HOSPITAL, 28 and 29 Southampton Street, Strand, London, W.C., and, to be in time for the current week's issue, should reach him by the first post on Mondays.

For Matrons' and Sisters' Appointments see page 268.

